# Pediatric Rapid Ultrasound for Shock and Hypotension Phenotype Differentiation in the Emergency Department: Evaluation of Feasibility and Reliability in a Malawi Cohort

**DOI:** 10.1097/PCC.0000000000003735

**Published:** 2025-03-31

**Authors:** Roxanne Assies, Yamikani Chimalizeni, Mercy Kumwenda, Harriet Khofi, Josephine Langton, Job B. M. van Woensel, Job C. J. Calis

**Affiliations:** 1 Department of Paediatrics and Child Health, Kamuzu University of Health Sciences, Blantyre, Malawi.; 2 Department of Paediatric Intensive Care, Amsterdam UMC, Location University of Amsterdam, Emma Children’s Hospital, Amsterdam, The Netherlands.; 3 Amsterdam Public Health, Global Health and Quality of Care, Amsterdam, The Netherlands.; 4 Department of Paediatrics and Child Health, Kamuzu Central Hospital, Lilongwe, Malawi.

**Keywords:** emergency department, low-resource setting, pediatric, point-of-care ultrasound, shock

## Abstract

**OBJECTIVES:**

To evaluate the feasibility, reliability, and diagnostic implications of performing the pediatric Rapid Ultrasound for Shock and Hypotension (p-RUSH) in children with undifferentiated shock upon hospital presentation in a low-resource setting (LRS).

**DESIGN:**

Prospective observational study from February 2019 to December 2019.

**SETTING:**

Pediatric emergency department (ED) of a large academic referral hospital in Blantyre, Malawi.

**PATIENTS:**

Children (2 mo to 16 yr old) with shock upon presentation to the pediatric ED.

**INTERVENTIONS:**

None.

**MEASUREMENTS AND MAIN RESULTS:**

Thirty children with shock were enrolled, of whom 14 died. The p-RUSH was performed upon admission to the ED, before administration of an IV fluid bolus. The p-RUSH was performed within a median time of 11.5 minutes, and 92.3% of the image frames in 4-second video clips were interpretable. Images were scored by two independent reviewers and the qualitative and quantitative assessments were compared and showed positive correlations as follows: 1) qualitative assessments of cardiac performance vs. left ventricle ejection fraction and fractional shortening measurements (*r* = 0.684 and *r* = 0.616, respectively, both *p* < 0.05) and 2) qualitative assessment of inferior vena cava (IVC) collapsibility vs. IVC collapsibility index (*r* = 0.470; *p* < 0.05). The interobserver agreement between cardiac and IVC qualitative assessments yielded a kappa statistic of up to 0.850 (cardiac views) and 0.275 (IVC collapsibility). Both reviewers applied a novel algorithmic flow diagram to diagnose the shock phenotype. In 23 of 30 children, the flowchart could be completed, which suggested either hypovolemic or distributive shock as the most common phenotype.

**CONCLUSIONS:**

In a Malawian pediatric ED, the p-RUSH was feasible and qualitative assessments were reliable. This 2019 proof-of-concept test provides a basis for further external validation of the p-RUSH and our algorithm for identifying shock phenotypes, which may lead to individualizing care of children presenting with shock in LRSs.

**RESEARCH IN CONTEXT**

- In a low-resource setting, such as Malawi, shock treatment for children presenting with fever and poor perfusion remains empirical and, typically, early IV fluid bolus is used.
- Point-of-care ultrasound (POCUS) may help distinguish the type of shock and lead to individualized interventions.
- In 2019, we used a structured POCUS protocol called the pediatric Rapid Ultrasound for Shock and Hypotension (p-RUSH) in our emergency department (ED) in Malawi to test the feasibility and reliability of the evaluations in children presenting with shock.

**AT THE BEDSIDE**

- In our 2019 study of 30 children in an ED in Malawi, the p-RUSH evaluation was feasible; qualitative assessment of cardiac function was reliable, but inferior vena cava collapsibility and diameter measurement were more difficult to interpret.
- Our proof-of-concept study, with a flow diagram algorithm combining the p-RUSH findings with hemoglobin concentration helped discern shock phenotype in critically ill children in the ED.
- Future clinical validation studies are required to assess individualized patient care.

Circulatory failure, or shock, is a life-threatening clinical syndrome and therefore important to recognize early in the assessment of critically ill children. Bolus administration of IV fluid is still considered the backbone of initial rapid treatment, but the 2009–2011 Fluid Expansion As Supportive Therapy (FEAST) randomized clinical trial (RCT) conducted in Eastern Africa showed that treating all children with febrile illness and clinical signs of shock with the same fluid administration protocol may be detrimental (1). Many questions remain regarding these findings, such as the diagnostic possibilities to determine the type of shock in this population and to subsequently individualize appropriate interventions. Furthermore, additional analyses of the FEAST-RCT data have also raised questions about optimal treatment in children with shock in low-resource settings (LRSs) (2). In this context, in 2018, we were aware that point-of-care ultrasound (POCUS) was used to better understand the diverse pathophysiology of shock and to individualize treatment in emergency and critical care (3, 4). In particular, there were specific POCUS protocols for adults (Rapid Ultrasound for Shock and Hypotension [RUSH]) and children with shock (pediatric RUSH [p-RUSH]) used to discriminate between different types of shock (5, 6). The p-RUSH may especially be useful in LRSs where diagnostic and treatment resources are limited and IV fluids should be administered with caution.

Therefore, in 2019, we aimed to assess the feasibility and reliability of an extended p-RUSH in children presenting to the emergency department (ED) with undifferentiated shock in a large hospital in Blantyre, Malawi. We also aimed to evaluate whether a structured algorithmic approach to p-RUSH data could be used to determine pediatric shock phenotype.

## MATERIALS AND METHODS

This prospective observational study was carried out in the resuscitation room of the pediatric ED of Queen Elizabeth Central Hospital (QECH) in Blantyre, Malawi, between February 2019 and December 2019. The study protocol was approved by the College of Medicine Research and Ethics Committee (COMREC P.12/18/2557) under the title “Circulatory insufficiency in sub-Saharan African Children” on January 7, 2019. Our center, QECH, is a large academic referral hospital where local triage and treatment protocols are based on the World Health Organization Emergency Triage Assessment and Treatment guidelines. Several consultant and trainee pediatricians at QECH were trained in applying POCUS, but performing POCUS (or the p-RUSH) was not standard of care in the ED. All study procedures were conducted in accordance with the ethical standards of COMREC and followed the principles outlined in the Helsinki Declaration of 1975.

We identified children in our ED who were 2 months to 16 years old and they were eligible for the study if they fulfilled the definition of shock used in the FEAST trial (1). That is at least one sign of shock, also associated with either: 1) impaired consciousness, defined as lethargy or a Blantyre Coma Scale of less than 5 or 2) respiratory distress, defined as increased work of breathing. The signs of shock were: capillary refill time greater than 3 seconds, cold peripheries, weak radial pulse, or severe tachycardia (defined as heart rate > 180/min if < 12 mo old, or > 160/min if age 1–5 yr, or > 140/min if 5–12 yr, or > 120/min if 12–16 yr old). Unlike the FEAST study: a) we did not exclude children with severe dehydration, malnutrition, or children without fever and b) we excluded children with an admission diagnosis of bronchiolitis, viral-induced wheeze, or asthma. If the child was eligible, verbal consent was gained from the parent or guardian, and written consent, in Chichewa or English, was gained as soon as possible and always within the first hour of admission to the ED resuscitation room. Children who required very urgent fluid resuscitation (i.e., within 15 min of presentation)—and therefore precluding our ability to obtain informed consent—were excluded from the study protocol. In practice, this did not lead to any exclusions of eligible children. The children included in the study were observed during the first hour in the ED resuscitation room and, in case of admission, followed up daily on the ward. Clinical care and decision-making was provided at the discretion of the ED staff irrespective of the ultrasound findings.

### p-RUSH and Data Collection

We performed the p-RUSH as proposed by Park et al (6). The p-RUSH was performed by a trained research physician (R.A.), using a Philips CX50 portable ultrasound machine (Philips, Amsterdam, The Netherlands) and two transducers: S5-1 broadband sector array transducer (5-1 MHz) and L12-3 broadband linear array transducer (12-3 MHz), which were provided for the study by Philips as a charitable offering. The p-RUSH was initiated prior to fluid bolus administration or blood transfusion in the ED. No sections of the p-RUSH were omitted. For this study, we performed an extended version of the p-RUSH, adding cardiac and inferior vena cava (IVC) measurements. We performed three cardiac views to assess cardiac function and two views of the IVC as a marker of circulatory status. Abdominal views as applied in the Focused Assessment with Sonography for Trauma (FAST) were made to assess the presence of abdominal free fluid, and lung ultrasound to assess for signs of pneumothorax, pleural effusion, or fluid overload. Details of the p-RUSH used in this study are described in **Supplementary Figure S1** (http://links.lww.com/PCC/C614). Ultrasound clips (frame rate in frames/s [fps], at 36–40 fps for 4 s) were saved and stored for evaluation by two reviewers (December 2019 to December 2020) who independently performed additional calculations including: left ventricular ejection fraction (LVEF), fractional shortening (FS), IVC collapsibility index (IVC-CI), and qualitative assessment on visual inspection of the ultrasound clips, using the same scoring questionnaire (Supplementary Fig. S1, http://links.lww.com/PCC/C614). Reviewer one (R1, R.A.) performed the ultrasound and reviewer two (R2, Y.C., pediatric cardiologist) scored the ultrasound images after receiving a short clinical description, excluding information about patient treatment or outcome. Based on a flow diagram using the p-RUSH findings and blood packed cell volume both reviewers determined which pathophysiological mechanism of shock was deemed the most likely in each patient, as we previously reviewed in 2008 (7).

### Statistical Analysis

We evaluated the p-RUSH feasibility by assessing the time needed to collect and store the images, and the interpretability of all images within the protocol. We evaluated the reliability of the p-RUSH by determining intraobserver and interobserver variability. For intraobserver variability between qualitative assessment and measured parameters of cardiac and IVC windows, we calculated the Pearson coefficient. For this analysis, we included the scores of both reviewers. Additionally, we reported the Pearson coefficient for each reviewer separately for IVC windows based on sensitivity analyses. Interobserver reliability of qualitative assessment of cardiac and IVC windows was assessed using the Pearson coefficient and weighted (squared) kappa coefficient. The maximum possible kappa value based on the observed marginal frequencies was calculated. Generally, a kappa coefficient of greater than 0.2–0.4 is interpreted as fair agreement, greater than 0.4–0.6 as moderate, greater than 0.6–0.8 as good, and greater than 0.8–1.0 as almost perfect agreement (8). We were not able to calculate reliability for lung and FAST windows because too few abnormal results were found in both. We used the Strengthening the Reporting of Observational Studies in Epidemiology recommendations to report our findings (9). Analysis and write-up of this article was carried out with delays as PhD related research tasks were paused due to clinical work during the COVID pandemic.

Continuous data are summarized as median and interquartile range (IQR). Numbers and proportions are presented for categorical data.

## RESULTS

In 2019, we enrolled 30 children with shock with a median age of 25.5 (IQR, 11.5–86.5) months. Fourteen children died (14/30). **Table 1** and **Supplementary Table S1** (http://links.lww.com/PCC/C614) summarize the demographics, clinical signs, laboratory results, and main discharge/death diagnosis. We were able to perform the p-RUSH in all 30 children and R1 found that in five of 30 overall cardiac contractility was reduced in the parasternal long axis (PLA) and subcostal views. IVC collapsibility was classified as greater than 50% collapse in 19 of 26, some collapse in four of 26, and no collapse in three of 26 (**Supplementary Fig. S2** and **Supplementary Table S2**, http://links.lww.com/PCC/C614). Signs of suprapubic free fluid were found in eight of 24 patients. No signs of pleural effusion or pneumothorax were found with lung ultrasound images.

**TABLE 1.** Demographic, Clinical, and Laboratory Results of Included Patients, Measured in the Emergency Department (*n* = 30)

Demographics	*n* or Median (IQR)
Age, mo	25.5 (IQR, 11.5–86.5)
Sex (male)	17/30
Weight (kg)^a^	10.0 (IQR, 7.6–19.2)
Mid upper arm circumference < 11.5 cm^b^	5/19
Inclusion criteria	
Reduced consciousness level^c^/lethargy	19/30
Respiratory distress^d^	23/30
Capillary refill time > 3 s	12/29
Cold peripheries	23/30
Weak pulse	17/30
Severe tachycardia^e^	22/30
Laboratory results	
Lactate	
> 2 mmol/L	22/28
> 5 mmol/L	13/28
Hemoglobin	
≤ 10 g/dL	14/30
≤ 5 g/dL	3/30
Hypoglycemia^f^	5/30
Hyperglycemia^g^	6/30
Positive blood culture^h^	2/22
Main discharge/death diagnosis	
Malaria	10/30
Gastroenteritis	9/30
Sepsis	4/30
Neurologic disease	3/30
Respiratory disease	1/30
Cardiac disease	1/30
Other	2/30
Outcome (died)	14/30

IQR = interquartile range.

a Weight estimated in four children with following formula: (age + 4) × 2.

b Mid upper arm circumference calculated for children 6–60 mo.

c Blantyre Coma Scale < 5.

d Auxiliary muscles and/or increased respiratory rate.

e Severe tachycardia was defined as > 180 beats/min if < 12 mo old, > 160 beats/min if 1–5 yr, > 140 beats/min if 5–12 yr, and > 120 beats/min if 12–16 yr old.

f Random blood sugar < 2.4 mmol/L or < 3.0 mmol/L for severely malnourished children.

g Random blood sugar > 10.0 mmol/L.

h One blood culture positive for *Escherichia coli* and one positive for *Staphylococcus aureus*.

### Feasibility

The p-RUSH could be performed in a median time of 11.5 minutes (range, 5.0–20.0 min) recording at least 22 clips per patient. Of all 960 images made, R1 and R2 reported 74 (7.7%) and 29 (3.0%) images as not interpretable, respectively. Cardiac and lung images were not interpretable in 27 of 420 (6.4%) and 15 of 240 (6.3%) images, respectively, compared with eight of 60 (13.3%) of IVC and 24 of 240 (10.0%) of FAST images (**Supplementary Table S3**, http://links.lww.com/PCC/C614).

### Reliability

**Figure 1.**
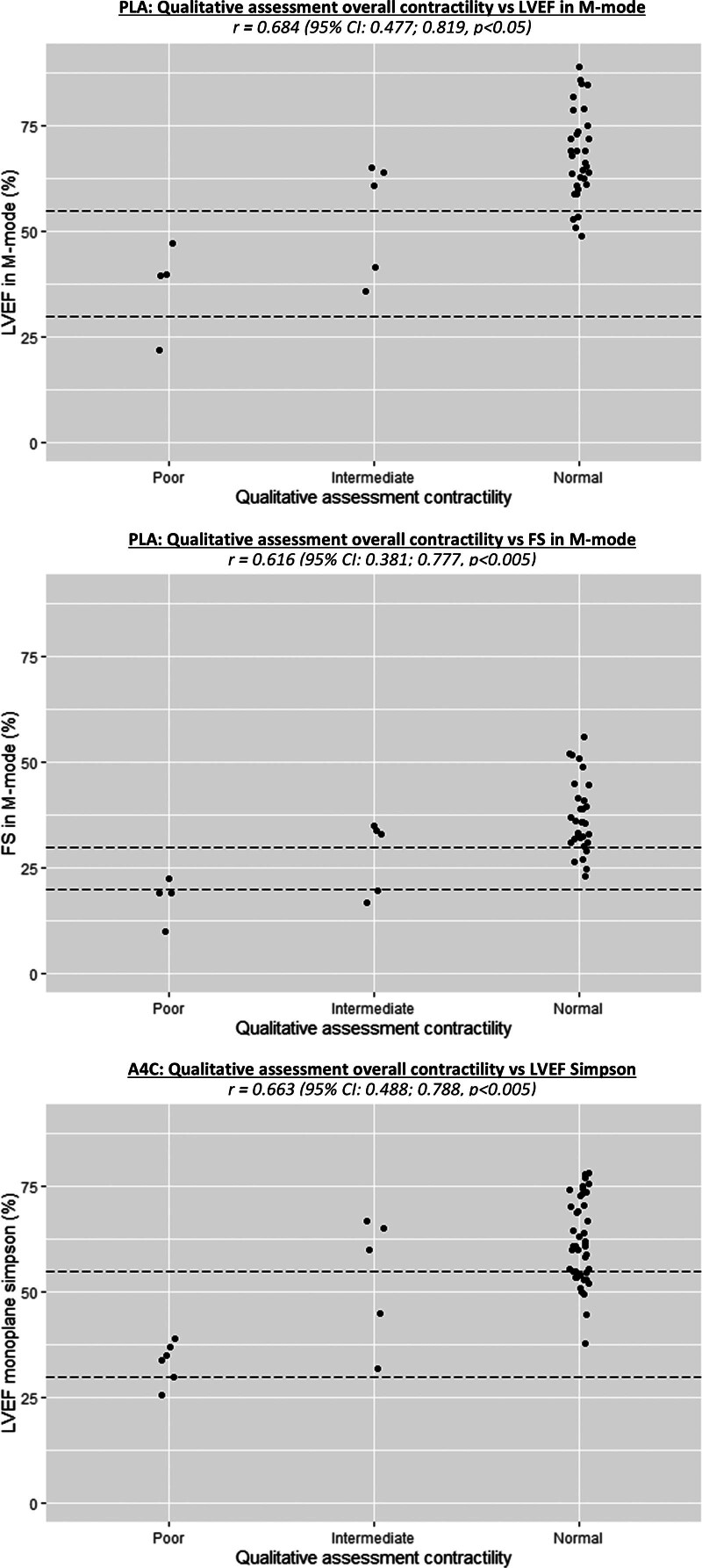
*Scatter plots* and correlation of cardiac qualitative vs. quantitative assessments. A4C = apical four chamber, FS = fractional shortening, LVEF = left ventricular ejection fraction, PLA = parasternal long axis.

**Figure 2.**
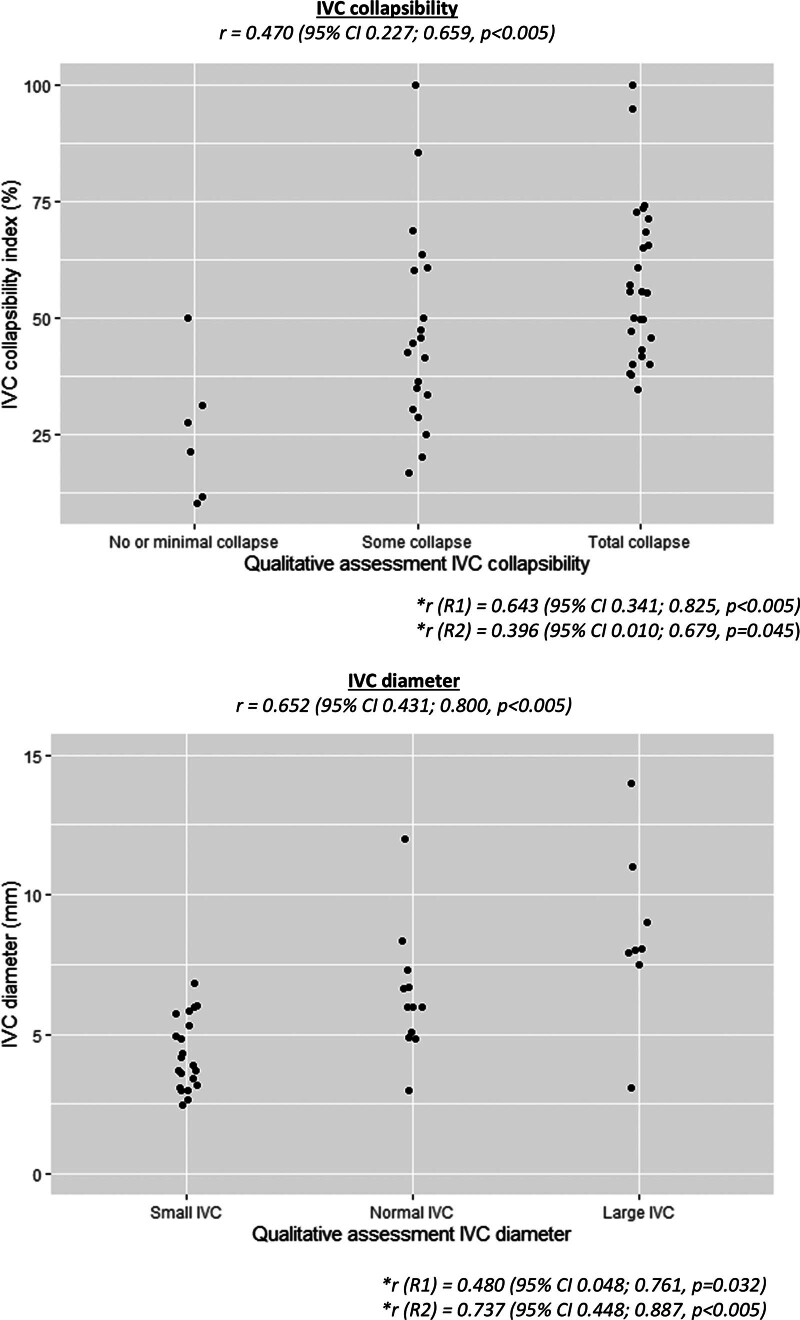
*Scatter plots* and correlation of inferior vena cava (IVC) qualitative vs. quantitative assessments.

Intraobserver agreement of qualitative assessment and measured parameters of cardiac contractility and IVC collapse or diameter, respectively, is shown in **Figures 1** and **2**. We found a positive correlation between qualitative assessment of cardiac contractility in the PLA and apical four chamber views compared with measured parameters. No patients with severely reduced cardiac contractility measured with LVEF or FS were interpreted as normal with qualitative assessment. We found positive correlations between qualitative assessment of IVC collapsibility and diameter with measured IVC-CI and measured IVC diameter. We found that all IVC diameters were smaller than 2 cm. **Table 2** describes the interobserver agreement for cardiac and IVC assessments. We found good interobserver agreement for qualitative assessment of cardiac contractility. For IVC collapsibility and IVC diameter, we found fair and good agreement, respectively.

**TABLE 2.** Interobserver Agreement of Qualitative Cardiac and Inferior Vena Cava Assessment

Ultrasound Assessment	Reviewer 1	Reviewer 2	Pearson Coefficient	Kappa^a^ and Maximum Kappa
Contractility parasternal long axis view	Poor: 3	Poor: 3	*r* = 0.624 (95% CI, 0.327–0.809; *p* < 0.005)	к = 0.623 (95% CI, 0.251–0.990; *p* < 0.005)
	Intermediate: 2	Intermediate: 3		Maximum к = 0.958
	Normal: 25	Normal: 22		
Contractility apical four chamber view	Poor: 4	Poor: 2	*r* = 0.652 (95% CI, 0.375–0.822; *p* < 0.005)	к = 0.628 (95% CI, 0.266–0.990; *p* < 0.005)
	Intermediate: 2	Intermediate: 3		Maximum к = 0.793
	Normal: 23	Normal: 25		
Contractility subcostal view	Poor: 3	Poor: 3	*r* = 0.858 (95% CI, 0.714–0.933; *p* < 0.005)	к = 0.850 (95% CI, 0.660–1.000; *p* < 0.005)
	Intermediate: 2	Intermediate: 1		Maximum к = 0.950
	Normal: 24	Normal: 25		
Size right ventricle and left ventricle apical four chamber view	LV < RV: 0	LV < RV: 0		к = 0.514 (95% CI, 0.037–0.990; *p* = 0.005)
	LV = RV: 4	LV = RV: 3		Maximum к = 0.838
	LV > RV: 25	LV > RV: 27		
IVC collapse	Total collapse: 19	Total collapse: 7	*r* = 0.340 (95% CI, –0.064 to 0.648; *p* = 0.097)	к = 0.275 (95% CI, 0.000–0.580; *p* = 0.089)
	Some: 4	Some: 16		Maximum к = 0.580
	No or minimal: 3	No or minimal: 3		
IVC diameter	Small: 17	Small: 13	*r* = 0.685 (95% CI, 0.398–0.850; *p* < 0.005)	к = 0.670 (95% CI, 0.390–0.950; *p* < 0.005)
	Normal: 5	Normal: 9		Maximum к = 0.802
	Large: 4	Large: 5		

IVC = inferior vena cava.

a Weighted (squared) kappa, except for level of agreement of right ventricle/left ventricle size (nominal instead of ordinal variable; therefore, we calculated unweighted kappa and no Pearson coefficient).

### Determination of Shock Phenotype

**Figure 3.**
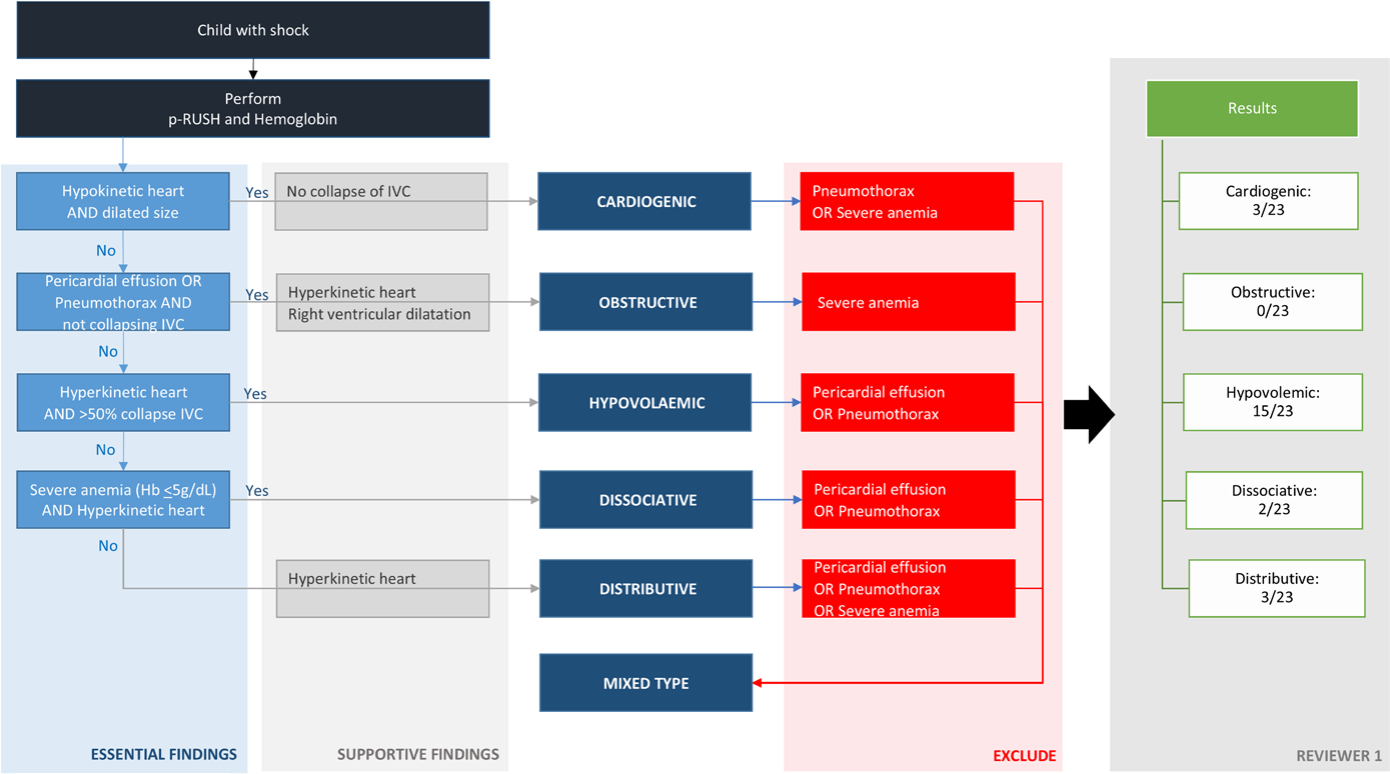
Flow diagram combining pediatric Rapid Ultrasound for Shock and Hypotension (p-RUSH) results. Hb = hemoglobin, IVC = inferior vena cava.

The flow diagram algorithm, combining different p-RUSH findings, could be applied in 23 of 30 children (**Fig. 3**). In seven children, windows that were needed to apply the algorithm could not be interpreted reliably. Hypovolemic shock was reported as the main underlying type of shock in 15 of 23 by R1; R2 concluded that distributive shock was the main underlying pathophysiological mechanism in 12 of 27 patients. Assessing interobserver agreement, we found a kappa statistic of 0.225 (*p* = 0.049), indicating fair agreement (**Supplementary Table S4**, http://links.lww.com/PCC/C614). Combining distributive and hypovolemic shock as one response, the kappa statistic was 0.526 (*p* < 0.001) indicating moderate agreement (**Supplementary Table S5**, http://links.lww.com/PCC/C614). We included an adapted version of the flow diagram as **Supplementary Figure S3** (http://links.lww.com/PCC/C614).

## DISCUSSION

In this 2019 study of 30 children managed in the ED in our hospital in Malawi, we have provided proof-of-concept that performing the p-RUSH was feasible, and the findings were reliable in children with shock in a LRS. The high early mortality of the 30 children underlined the importance of improved ED treatment guidelines. This study also demonstrates that the p-RUSH could be performed reliably in under 20 minutes (median time < 12 min). Both of our reviewers showed that qualitative assessment could reliably determine heart contractility; however, IVC collapsibility was more difficult to interpret. This finding was also reflected in our proposed p-RUSH flow diagram, in which hypovolemic or distributive shock was identified as the main type of shock, depending on the reviewer. These findings provide important results that the p-RUSH has potential to help guide treatment in a LRS, especially with improved assessment of IVC collapsibility.

Our first message is that the p-RUSH provides reliable qualitative assessment of cardiac function. For example, we found that qualitative assessment of left ventricle contractility correlated with FS and LVEF measurements, and there was good interobserver agreement in the qualitative assessment. This observation is consistent with other studies reporting that qualitative cardiac assessment is a reliable method in pediatric and adult emergency settings, in both low- and high-resource settings (10–13).

Second, we found that IVC diameter and collapsibility may be more difficult to interpret. The same limitation applies to the IVC-CI; although in a cohort of patients with malaria (both children and adults) in Gabon, studied in 2019, an IVC-CI of greater than or equal to 50% was associated with clinical signs related to hypovolemia (14). For IVC/aorta ratio, both of our reviewers found similar values, which are like the results of a 2006–2007 study of 36 children with dehydration (15). We found good interobserver agreement for qualitative assessment of IVC diameter, but a less, albeit, still fair agreement for IVC collapsibility. If we take account of information provided in a 2020 narrative review about pediatric studies on IVC collapsibility and distensibility indices (16), along with our own current 2019 work, we conclude that qualitative IVC assessment may be reliable. However, such assessment may be improved by sequential assessment or by a second clinician when there is doubt about the findings.

The flow diagram, presented as Figure 3 in this report, was developed to help distinguish between five different types or phenotypes of shock (i.e., hypovolemic, obstructive, cardiogenic, distributive, or dissociative). We added the patient’s hemoglobin concentration to this flow diagram because it is especially relevant in sub-Saharan Africa where the prevalence of anemia is high, and this type of shock requires a different approach to treatment, such as blood transfusion (1). Either hypovolemic or distributive shock were the most reported type of shock, depending on our reviewer making the assessment. When one reviewer scored hypovolemic shock, the other more often scored this as distributive shock and vice versa. This discrepancy is similar to observations summarized in a systematic review of the literature through to 2019, in which the reviewers reported that hypovolemic as well as distributive shock were the most prevalent types of shock. Furthermore, the authors of the review acknowledged the difficulty in distinguishing between hypovolemic and distributive shock from a cardiac perspective because in both, circulatory volume is decreased either due to blood or fluid loss, or vasodilation, respectively, or a combination of these factors like in septic shock (17). The practical implications of these differences may be purely academic, rather than important for clinical practice, since treatment advised by an international group of experts in a 2023 viewpoint article stated that a fluid bolus followed by inotropes may be applied in both situations, in the absence of cardiac malfunction (18). A previous prospective study in a pediatric ward in Malawi, 2017–2018, applied focused cardiac ultrasound (FOCUS) to determine cardiac dysfunction in 54 children with severe febrile illness and impaired circulation and found impaired left ventricular function in a quarter of the children (19). This previous study corroborates findings in our study. In fact, we adapted the flow diagram after analyzing our results, taking into consideration the challenge of interpreting IVC measurements and distinguishing between hypovolemic and distributive shock. However, further studies are needed to better evaluate and optimize the flow diagram and address the interobserver variability and to guide treatment of pediatric shock in LRSs.

The limitations of our study includes its small sample size, p-RUSH performed by a single clinician, and limited diagnostic resources. First, to determine the intraobserver and interobserver reliability with smaller CIs, a larger sample size would have been preferable. The interobserver reliability of cardiac function may have been underestimated due to different training level and background of the reviewers; R1 was a medical doctor working in the ED, R2 was a cardiologist, but both were trained in POCUS. Furthermore, R2 had to score the p-RUSH with a brief clinical history and description of the patient, which may have influenced interpretation of the p-RUSH findings and affected the application of the flow diagram. This procedure may however represent the clinical setting, in which it would be implemented. Lastly, we performed this study in a LRS where diagnostic resources are limited, and apart from a blood gas and hemoglobin measurement, we did not perform extra diagnostic testing. We recruited children based on clinical signs according to the FEAST criteria. Although these criteria may be less specific, this aligns with clinical practice in African settings and the high prevalence of lactatemia underscores the critical severity of illness of our population.

Application of the p-RUSH may have impact in a LRS, like Malawi, where pediatric shock is a common clinical syndrome with high mortality, while diagnostic and human resources to further guide and monitor treatment are scarce. Therefore, the systematic approach described in the p-RUSH may be helpful to guide and individualize treatment. Although the application of the p-RUSH for children with undifferentiated shock is new, only a few studies have proposed POCUS/FOCUS assessments as measures to guide and monitor treatment of children with shock (18, 20). We do see the value of determining the hemoglobin concentration (7), especially in regions with a high prevalence of anemia and malaria, and of performing lung and FAST views when deemed relevant based on history and/or physical examination. Implementation of the p-RUSH would depend on the availability of ultrasound equipment and training; however, this technology is increasingly available in a wide variety of settings (21, 22), and assistance of artificial intelligence may now be applied to improve quality of images (23–25). In this context, our results provide a starting point for further research to improve understanding of shock and move towards improved treatment. This program of work should include evaluating whether the p-RUSH can also be used to monitor response to treatment, the impact on clinical decision-making in the ED, and any consequence on morbidity and mortality in children presenting with shock in an LRS (26). In our ED in Blantyre, POCUS is widely used as medical staff are increasingly trained and ultrasound machines are more readily available. Development and publication of the p-RUSH algorithm will help to systematically apply POCUS findings into clinical practice for children with shock (27, 28).

In conclusion, our 2019 study in children presenting with shock in our ED in Malawi shows that performing the p-RUSH was feasible during the first hour of presentation. Cardiac assessments were reliable, but the interpretation of IVC collapsibility appeared to be more difficult to assess. Taken together, as a proof-of-concept, we believe that a structured flow diagram helps with determining the type of shock, but further studies are needed to validate this approach.

## ACKNOWLEDGMENTS

We thank the children and their guardians for participating in this study. Furthermore, we thank the nurses and doctors who cared for these children at Queen Elizabeth Central Hospital. Lastly, we thank Dr. Kuipers, a pediatric cardiologist at Amsterdam University Medical Center, for her input and training of personnel involved in the project.

## Supplementary Material

SUPPLEMENTARY MATERIAL
