# Supplementary material for: Pediatric Rapid Ultrasound for Shock and Hypotension Phenotype Differentiation in the Emergency Department: Evaluation of Feasibility and Reliability in a Malawi Cohort
Source: Pediatr Crit Care Med. 2025 Mar 31;26(7):e867–76. doi: 10.1097/PCC.0000000000003735 (PMC12212470; doi:10.1097/PCC.0000000000003735)
Supplement: SUPPLEMENTARY MATERIAL [file pcc-26-e867-s001.docx]

**Table of contents**

Supplementary Figure S1: p-RUSH protocol and checklist for reviewers 2-9

Supplementary Figure S2: Selection of p-RUSH images of two included patients 10

Supplementary Table S1: Additional clinical and laboratory results of included patients 11

Supplementary Table S2: Overview ultrasound scoring questions reviewer one and two 12-14

Supplementary Table S3: Ultrasound views scored as not interpretable 15

Supplementary Table S4: Type of shock reported by reviewer one and two 16

Supplementary Table S5: Interobserver reliability type of shock, hypovolemic/distributive
combined 16

Supplementary Figure S3: Adapted flow diagram 17

**Figure S1: p-RUSH protocol**

| Patient ID |  |
| --- | --- |
| Patient date of birth (dd/mm/yy) |  |
| Patient length (cm) |  |
| Patient weight (kg) |  |
| Date (dd/mm/yy) |  |
| Time start |  |
| Time finished |  |
| Reviewer: |  |

**General**

- Equipment needed: Ultrasound machine (CX50), probes (cardiac, abdominal and linear), gel, wipes
- Patient in supine position. If not, please specify position and why: …………………………………………………………………………………………………….
- Fill in patient ID
- Indicator should be on the right side of the screen for the cardiac and IVC windows and on the left side of the screen for the FAST and lung windows
- Perform p-RUSH with following windows, instructions and scoring

**Pump (Heart)**

- Components to assess: contractility of the heart, pericardial effusion, tamponade
- Probe: Phased array transducer (Cardiac probe S5-1)

Windows:

**Parasternal Long Axis view**

**Apical four chamber view**

**Subxiphoid view**

| **INDICATOR OF THE SCREEN IS ON THE RIGHT** |
| --- |

| - 1. **Parasternal long axis view** - Position probe: left of the sternum, 3-4rd intercostal space, indicator pointing towards the patient’s right shoulder - Save one 10 sec clip in 2D mode (Clip 1) - Save one image in M-mode (Clip 2) | |
| --- | --- |
| Is there any pericardial effusion? | - Yes - Minor (approx. <15 mm) - No - Cannot interpret   Comments  ………………………………………….. |
| Are there any signs of tamponade?  *Tick which signs apply* | - No signs - Circumferential pericardial effusion - Diastolic collapse right ventricle - “Oscillating” or “dancing” heart - Cannot interpret   Comments …………………………………………… |
| How is the heart rate?  *Through eyeballing, considering age of the patient* | - Normal - Hyperkinetic (heart rate is fast) - Hypokinetic (heart rate is slow) - Cannot interpret   Comments  ………………………………………….. |
| How is the contractility of the left ventricle?  *Assessed through eyeballing/ visual assessment of the following three components:* | |
| 1. How is the change in size of the left ventricle (squeeze of the septal wall to the posterior left ventricle wall) | - Normal - Intermediate - Poor - Cannot interpret   Comments ………………………………………….. |
| 1. How is the thickening of the posterior left ventricle wall during squeeze of the heart | - Normal - Intermediate - Poor - Cannot interpret   Comments ………………………………………….. |
| 1. How is the anterior mitral leaflet movement? | - Normal - Intermediate - Poor - Cannot interpret   Comments ………………………………………….. |
| 1. Overall assessment contractility | - Normal - Intermediate - Poor - Cannot interpret   Comments ………………………………………….. |
| The following measurement should be done AFTER initial p-RUSH from the saved image | |
| Left ventricular ejection fraction estimation  *M-mode line over distal tip of anterior mitral valve and calculate using Teichholz method.* | ……… % |
| Fractional shortening  *Calculated in M-mode using LVESD and LVEDD* | ………. % |
| What is the quality of the window? | - Good - Fair - Poor   Comments  ……………………………………………. |

| **Apical four chamber view**   - Position probe: apex of the heart, indicator pointing towards the left shoulder of the patient - Save one 10 second clip (Clip 3) | |
| --- | --- |
| Is there any pericardial effusion? | - Yes - Minor (approx. <15 mm) - No - Cannot interpret   Comments  ………………………………………….. |
| Are there any signs of tamponade?  *Tick which signs apply* | - No signs - Circumferential pericardial effusion - Diastolic collapse right ventricle - “Oscillating” or “Dancing” heart - Cannot interpret   Comments …………………………………………… |
| How is the heart rate?  *Through eyeballing, considering age of the patient* | - Normal - Hyperkinetic (heart rate is fast) - Hypokinetic (heart rate is slow) - Cannot interpret   Comments  ………………………………………….. |
| How is the contractility of the left ventricle?  *Assessed through eyeballing* | - Normal - Intermediate - Poor - Cannot interpret   Comments ………………………………………….. |
| Compare the size of the right and left ventricle: | - Right ventricle > left ventricle - Right ventricle < left ventricle - Right ventricle = left ventricle - Cannot interpret   Comments …………………………………………….. |
| The following measurement should be done AFTER initial p-RUSH from the saved clips | |
| What is the RV/LV ratio?  *Normal ratio 0.6:1* | RV…………/ LV……………. |
| Left ventricular ejection fraction estimation U*sing the Simpson method* | ……… % |

| **Subxiphoid window**   - Position probe: transverse position with the indicator to the patient’s left side, inferior of the xyphoid, probe slightly angled towards the patient’s left scapula - Save one 10 second clip (Clip 4) | |
| --- | --- |
| Is there any pericardial effusion? | - Yes - Minor (approx. <15 mm) - No - Cannot interpret   Comments  ………………………………………….. |
| Are there any signs of tamponade?  *Tick which signs apply* | - No signs - Circumferential pericardial effusion - Diastolic collapse right ventricle - “Oscillating” or “Dancing” heart - Cannot interpret   Comments …………………………………………… |
| How is the heart rate?  *Through eyeballing, considering age of the patient* | - Normal - Hyperkinetic (heart rate is fast) - Hypokinetic (heart rate is slow) - Cannot interpret   Comments  ……………………………………. |
| How is the contractility of the left ventricle?  *Assessed through eyeballing* | - Normal - Intermediate - Poor - Cannot interpret   Comments ………………………………………….. |
| Compare the size of the right and left ventricle: | - Right ventricle > left ventricle - Right ventricle < left ventricle - Right ventricle = left ventricle - Cannot interpret   Comments …………………………………………….. |
| What is the quality of the window? | - Good - Fair - Poor - Cannot interpret   Comments  ……………………………………………. |

**Tank**

- Components to assess: IVC, thoracic and abdominal compartment (abdominal FAST views)
- Probe: phased array transducer (Cardiac probe S1-5). In larger children consider switching to a curvilinear probe (Abdominal probe)

Windows:

- 1. **Subxiphoid**
  2. **RUQ**
  3. **LUQ**
  4. **Suprapubic**

| - 1. **Subxiphoid window: IVC** - View 1: Transverse position of the probe, probe placed subxiphoid position with the indicator to patient’s left side - View 2: Longitudinal/Sagittal position of the probe: probe placed subxiphoid position with indicator towards patient’s head, including M-mode. IVC examination 2-3 cm caudal of the right atrium. - Save a 5 sec clip of view 1 (Clip 5) - Save a 10 sec clip of view 2 (Clip 6) - Save one image of M-mode in view 2 (Clip 7) | |
| --- | --- |
| Is there any IVC collapse with inspiration?  *Assessed through eyeballing in longitudinal view and M-mode view* | - Yes, total collapse - Yes, some collapse - No or minimal collapse - Cannot interpret - Comments   …………………………………………………. |
| What is the diameter of IVC  *Assessed through eyeballing in longitudinal view* | - Normal - Small - Large - Cannot interpret - Comments   …………………………………………………. |
| The following measurement should be done AFTER initial p-RUSH from the saved clips | |
| What is the IVC/Aorta ratio (IVC/AO ratio)? *Measure in transverse view using maximum anterior-posterior diameter of the IVC and descending Aorta during expiration.* | Diameter IVC:……………….mm  Diameter Aorta:……………mm  IVC/AO ratio: IVC………../Ao…………. |
| What is the Collapsibility index (CI)?  *Measure in clip of longitudinal view. Measure maximum and minimum diameter of IVC and calculate CI.* | Min. diameter CI…………..mm  Max. diameter CI………….mm  ………………..% |
| What is the quality of the window? | - Good - Fair - Poor   Comments  ……………………………………………. |

| **SWITCH INDICATOR OF THE SCREEN TO THE LEFT** |
| --- |

| **Right Upper Quadrant (RUQ)**   - Position probe along the right upper quadrant mid axillary line with indicator towards patient’s head and visualize the liver, the caudal tip of the liver, kidney, Morison’s pouch and diaphragm. - Save three 10 sec clips:   - One of the diaphragm (Clip 8)   - One of Morison’s pouch (Clip 9)   - One of the caudal tip of the liver (Clip 10) | |
| --- | --- |
| Is there any pleural effusion? | - Yes* - No - Cannot interpret   Comments  ………………………………………………….  * no lung curtain sign or positive spine sign or floating lung tip in effusion |
| Is there any free fluid in Morison’s pouch? | - Yes - No - Cannot interpret   Comments  …………………………………………………. |
| Is there any free perihepatic fluid (caudal tip liver)? | - Yes - No - Cannot interpret   Comments  …………………………………………………. |
| What is the quality of the window? | - Good - Fair - Poor   Comments  ……………………………………………. |

| **Left Upper Quadrant (LUQ)**   - Position the probe along the left upper quadrant in the mid axillary line with indicator towards the head of the patient and visualize the spleen, kidney and diaphragm - Save two 10 sec clips:   - One of the diaphragm (Clip 11)   - One of the spleen and kidney (splenorenal recess) (Clip 12) | |
| --- | --- |
| Is there any pleural effusion? | - Yes* - No - Cannot interpret - Comments   ………………………………………………….  * no lung curtain sign or positive spine sign or floating lung tip in effusion |
| Is there any perisplenic* free fluid?  * between spleen and diaphragm | - Yes - No - Cannot interpret - Comments   …………………………………………………. |
| Is there any free fluid in the splenorenal recess? | - Yes - No - Cannot interpret - Comments   …………………………………………………. |
| What is the quality of the window? | - Good - Fair - Poor   Comments  ……………………………………………. |

| **Suprapubic window**   - Position the probe suprapubic in the transverse view (indicator towards the right side of the patient) and then the longitudinal position (indicator towards the head of the patient) to visualize the bladder - Save two 10 sec clips:   - One of the transverse view of the bladder (Clip 13)   - One of the longitudinal/sagittal view of the bladder (Clip 14) | |
| --- | --- |
| Is there any free fluid posterior to the bladder (transverse view)? | - Yes - No - Cannot interpret - Comments   …………………………………………………. |
| Is there any free fluid posterior to the bladder (longitudinal view) | - Yes - No - Cannot interpret - Comments   …………………………………………………. |
| What is the quality of the window? | - Good - Fair - Poor   Comments  ……………………………………………. |

**Lungs**

- Components to assess: pneumothorax of the left and right lung, signs of pulmonary edema
- Probe: change to linear probe

Windows:

**Right lung, anterior view (including M-mode)**

**Right lung, lateral view (including M-mode)**

**Left lung, anterior view (including M-mode)**

**Left lung, lateral view (including M-mode)**

| - 1. **Right lung – anterior view** - Position the probe in longitudinal position on the most anterior point of the thorax right of the sternum with the indicator towards the head of the patient - Save a 5 sec clip in 2D mode (Clip 15) - Save one image in M-mode (Clip 16) | |
| --- | --- |
| Is there any lung sliding? | - Yes - No - No (pleural effusion) - Cannot interpret - Comments   …………………………………………………. |
| Are there any B-lines? | - No - Yes, <3/ICS - Yes, >3/ICS (B-pattern) - Confluent B-lines - White lung - Cannot interpret - Comments   …………………………………………………. |
| What is the quality of the window? | - Good - Fair - Poor   Comments  ……………………………………………. |

| - 1. **Right lung - lateral view** - Position the probe in longitudinal position as posterior as possible (at least on the posterior axillar line) on the right side of the patient with the indicator towards the head of the patient - Save a 5 sec clip in 2D mode (Clip 17) - Save one image in M-mode (Clip 18) | |
| --- | --- |
| Is there any lung sliding? | - Yes - No - No (pleural effusion) - Cannot interpret - Comments   …………………………………………………. |
| Are there any B-lines? | - No - Yes, <3/ICS - Yes, >3/ICS (B-pattern) - Confluent B-lines - White lung - Cannot interpret - Comments   …………………………………………………. |
| What is the quality of the window? | - Good - Fair - Poor   Comments  ……………………………………………. |

| **Left lung – anterior view**   - Position the probe in longitudinal position on the most anterior point of the thorax left of the sternum with the indicator towards the head of the patient - Save a 5 sec clip in 2D mode (Clip 19) - Save one image in M-mode (Clip 20) | |
| --- | --- |
| Is there any lung sliding? | - Yes - No - No, pleural effusion - Cannot interpret - Comments   …………………………………………………. |
| Are there any B-lines? | - No - Yes, <3/ICS - Yes, >3/ICS (B-pattern) - Confluent B-lines - White lung - Cannot interpret - Comments   …………………………………………………. |
| What is the quality of the window? | - Good - Fair - Poor   Comments  ……………………………………………. |

| - 1. **Left lung – lateral view** - Position the probe in longitudinal position as posterior as possible (at least on the posterior axillar line) on the right side of the patient with the indicator towards the head of the patient - Save a 5 sec clip in 2D mode (Clip 21) - Save one image in M-mode (Clip 22) | |
| --- | --- |
| Is there any lung sliding? | - Yes - No - No, pleural effusion - Cannot interpret - Comments   …………………………………………………. |
| Are there any B-lines? | - No - Yes, <3/ICS - Yes, >3/ICS (B-pattern) - Confluent B-lines - White lung - Cannot interpret - Comments   …………………………………………………. |
| What is the quality of the window? | - Good - Fair - Poor   Comments  ……………………………………………. |

**Assessment type of shock**

**Can the type of shock be determined with the p-RUSH findings?**

- Yes
- No

**What type of shock is most likely based on the p-RUSH findings?**

- Hypovolemic shock
- Distributive shock
- Obstructive shock
- Cardiogenic shock
- Dissociative shock

**Supplementary Figure 1: p-RUSH protocol describing how to perform the different ultrasound views, how to score the views through qualitative assessment (‘eyeballing’) and what measurements to perform.**

**
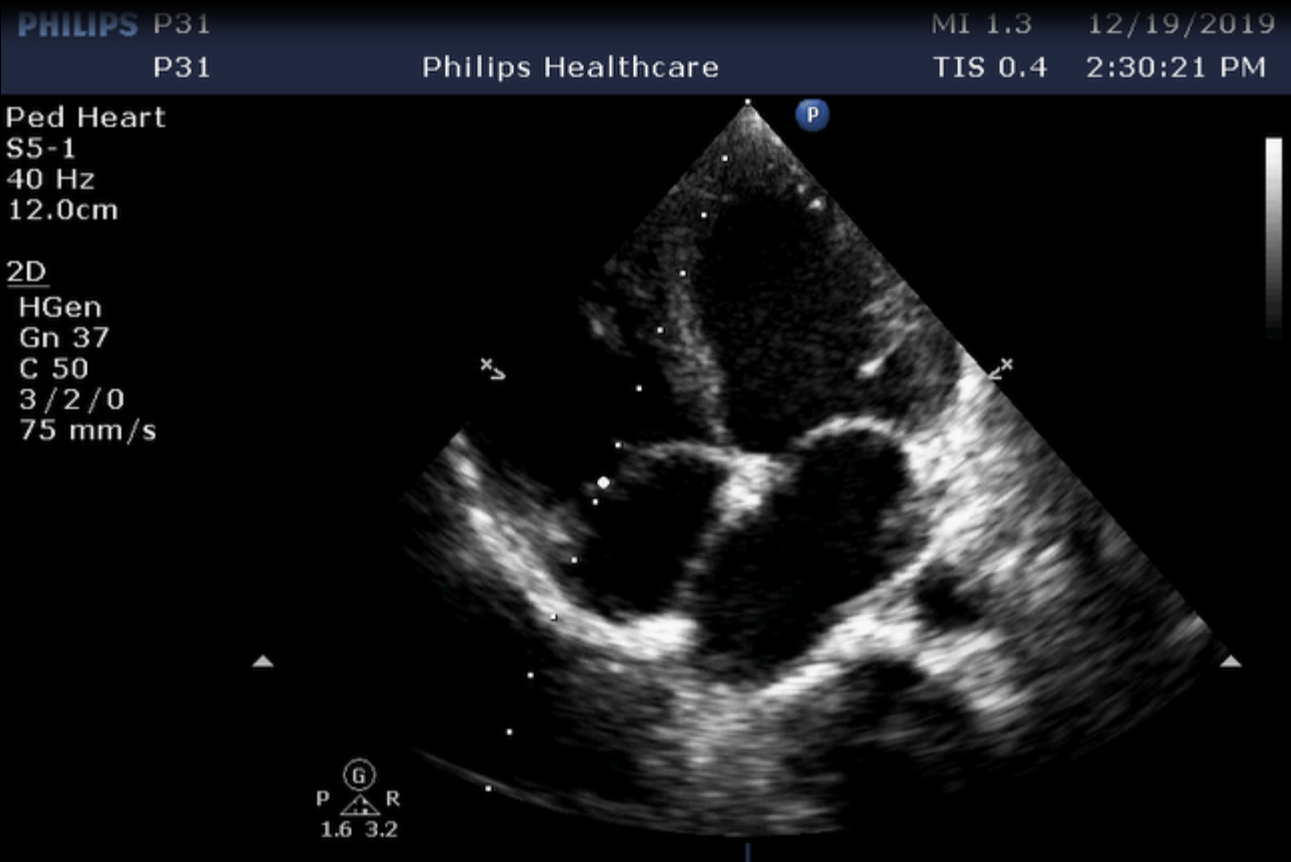

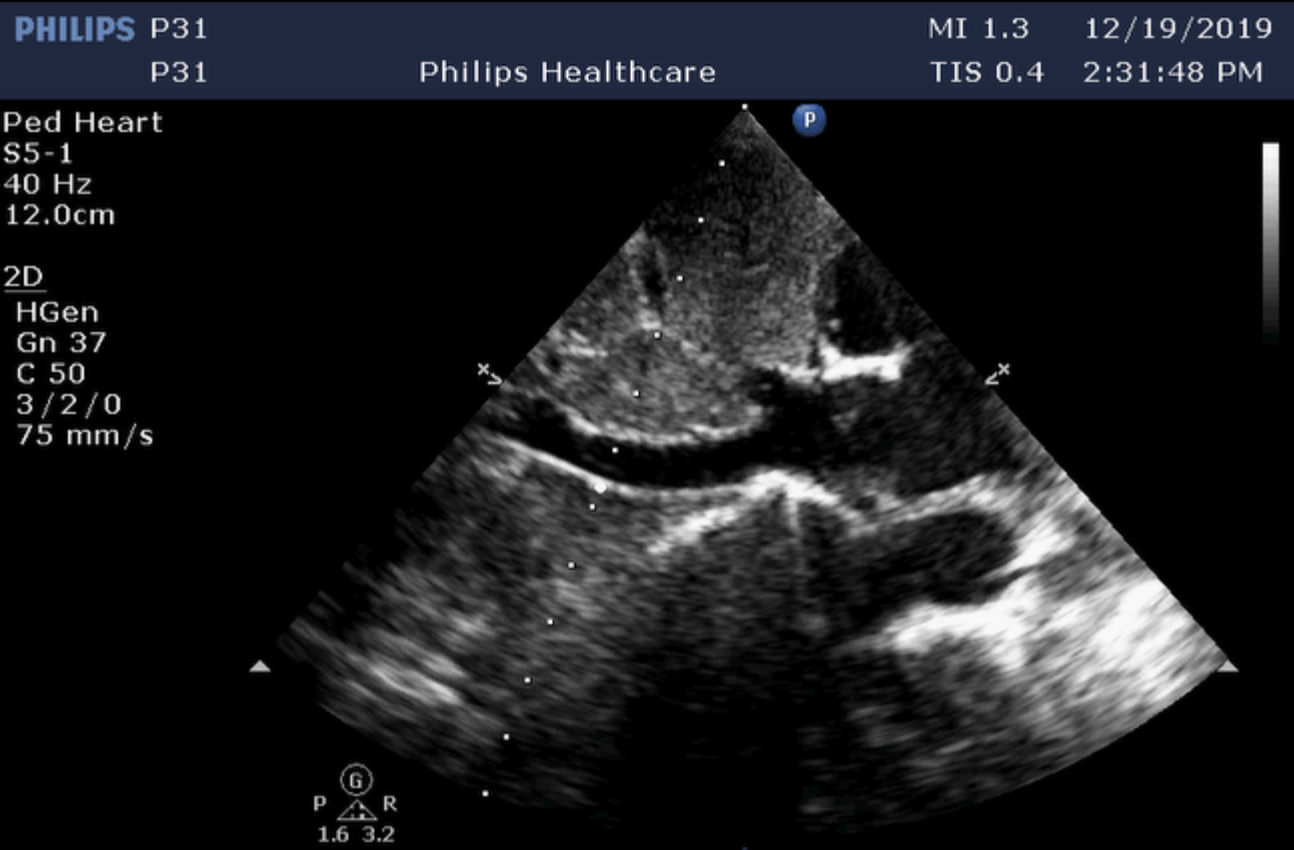

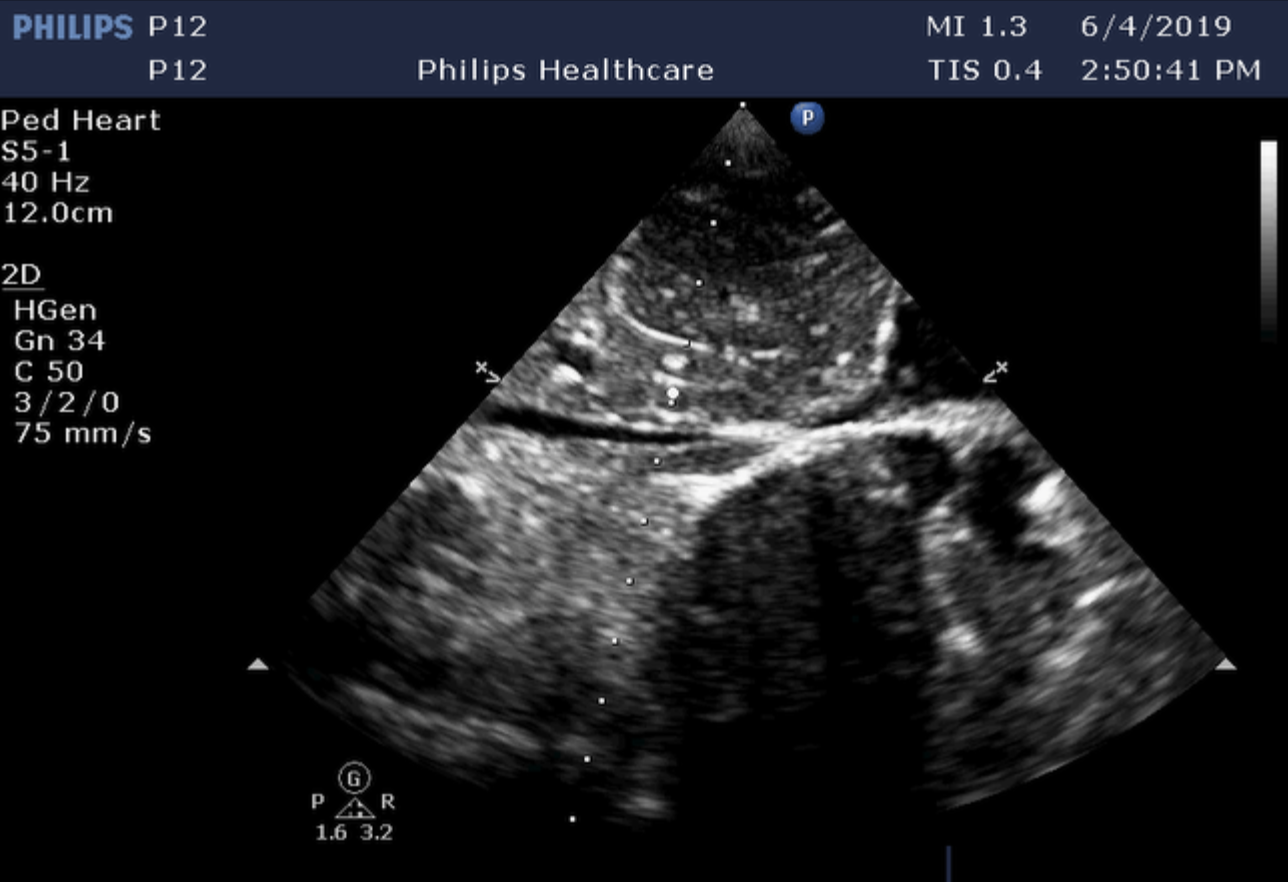

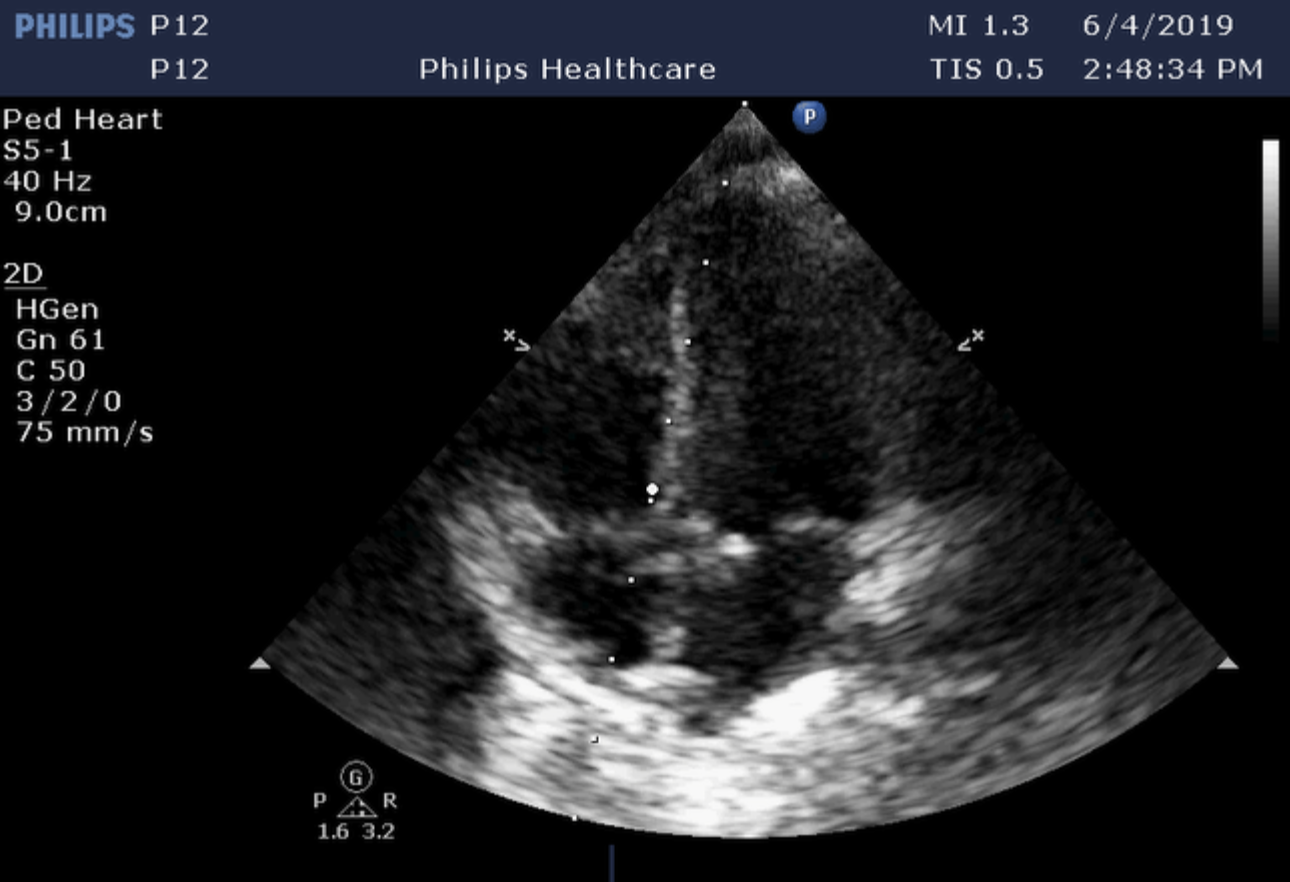
**

**Supplementary Figure S2: Selection of p-RUSH images of two included patients. On the top left the apical four chamber (A4C) view showing a dilated heart, reduced overall contractility (on clip) and a dilated inferior vena cava (IVC) on the subcostal (IVC) view in the bottom left image with no/minimal collapse (on clip). On the top right the A4C view with a normal heart with good overall contractility (on clip) with a small and collapsing (>50%) IVC on the subcostal (IVC) view on the bottom right image (and on clip).**

**Supplementary Table S1: Additional clinical and laboratory results of included patients, measured in the emergency department (N=30)**

| **Clinical signs** | **n or median (IQR)** |
| --- | --- |
| Respiratory rate | 53 (IQR: 41 – 69) |
| SpO2 | 97 (IQR: 96 – 100) *5 missing |
| Heart rate | 161 (IQR: 137 – 185) |
| SBP | 98 (IQR: 94 – 105) *8 missing |
| DBP | 60 (IQR: 48 – 67) *9 missing |
| CRT | 3 (IQR: 2 – 4) *1 missing |
| BCS | 4 (IQR: 2 – 5) |
| Temperature >38.0°C | 18/29 |
| **Laboratory results** |  |
| MPS/MRDT positive | 11/30 |
| HIV  Positive  Exposed* | 4/30  2/30 |
| **Outcome (died)** | 14/30 |
| <24 hours | 8/14 |
| <48 hours | 12/14 |

*Children <1 year of age with HIV positive mother and tested negative for HIV, missing for 6 children

Abbreviations: BCS= Blantyre Coma Scale, CRT= capillary refill time, SBP= Systolic Blood Pressure, DBP= Diastolic Blood Pressure, MPS= Malaria Parasites (blood smear), MRDT= Malaria Rapid Diagnostic Test, HIV= Human Immunodeficiency Virus

**Supplementary Table S2: Overview of all scoring questions for the ultrasound views for reviewer 1 and reviewer 2.**

|  | **R1** | | **R2** | |
| --- | --- | --- | --- | --- |
|  | **Cannot interpret** | **Findings** | **Cannot interpret** | **Findings** |
| **Parasternal long axis** | **3** |  | **7** |  |
| - Pericardial effusion? | 2 | Yes: 0  Minor: 4  No: 24 | 2 | Yes: 0  Minor: 6  No: 22 |
| - Tamponade? | 1 | Yes: 0  No: 29 | 2 | Yes: 0  No: 28 |
| - Heart rate? | 0 | Hypokinetic: 2  Hyperkinetic: 23  Normal: 5 | 1 | Hypokinetic: 1  Hyperkinetic: 25  Normal: 3 |
| - Change in size of the left ventricle? | 0 | Poor: 4 Intermediate: 1 Normal: 25 | 3 | Poor: 3 Intermediate: 3 Normal: 21 |
| - Thickening of the posterior left ventricle wall? | 1 | Poor: 3 Intermediate: 2 Normal: 24 | 2 | Poor: 2 Intermediate: 1 Normal: 25 |
| - Anterior mitral leaflet movement? | 1 | Poor: 2 Intermediate: 0 Normal: 27 | 2 | Poor: 1 Intermediate: 1 Normal: 26 |
| - Overall contractility? | 0 | Poor: 3  Intermediate: 2  Normal: 25 | 2 | Poor: 3  Intermediate: 3  Normal: 22 |
| - LVEF (M-mode) | 11 | Median: 63.8  IQR: 52.3 – 67.7  Poor (<30): 0 Intermediate (30-55): 6  Normal (>55): 13 | 7 | Median: 68.0 IQR: 60.0 – 74.0  Poor (<30): 1 Intermediate (30-55): 3 Normal (>55): 19 |
| - FS (M-mode) | 11 | Median: 32.4 IQR: 25.7 – 35.8  Poor (<20): 2  Intermediate (20-30): 3 Normal (>30): 13 | 7 | Median: 36.0 IQR: 31.5 – 40.0  Poor (<20): 1  Intermediate (20-30): 3 Normal (>30): 19 |
| **Apical four chamber** | **6** |  | **1** |  |
| - Pericardial effusion? | 3 | Yes: 0  Minor: 5  No: 22 | 0 | Yes: 1  Minor: 3  No: 26 |
| - Tamponade? | 1 | Yes: 0  No: 29 | 0 | Yes: 0  No: 30 |
| - Heart rate? | 0 | Hypokinetic: 2  Hyperkinetic: 24  Normal: 4 | 1 | Hypokinetic: 1  Hyperkinetic: 27  Normal: 1 |
| - Contractility LV? | 1 | Poor: 4  Intermediate: 2  Normal: 23 | 0 | Poor: 2  Intermediate: 3  Normal: 25 |
| - LVEF (Simpson) | 1 | Median: 60.0  IQR: 50.2 – 70.3  Poor (<30): 2 Intermediate (30-55): 9 Normal (>55): 18 | 2 | Median: 60.5 IQR: 53.0 – 65.5  Poor (<30): 0 Intermediate (30-55): 12 Normal (>55): 16 |
| - Size RV and LV | 1 | LV < RV: 0  LV = RV: 4  LV > RV: 25 | 0 | LV < RV: 0  Right=left: 3  Left>right: 27 |
| - RV/LV ratio | 3 | Median: 0.87 IQR: 0.81 – 0.96 | 3 | Median: 0.75  IQR: 0.68 – 0.83 |
| **Subcostal** | **18** |  | **5** |  |
| - Pericardial effusion? | 1 | Yes: 0 Minor: 10 No: 19 | 1 | Yes: 0 Minor: 9 No: 20 |
| - Tamponade? | 1 | Yes: 0 No: 29 | 1 | Yes: 0 No: 29 |
| - Heart rate? | 0 | Hypokinetic: 2 Hyperkinetic: 22 Normal: 6 | 0 | Hypokinetic: 1 Hyperkinetic: 27 Normal: 2 |
| - Contractility LV? | 1 | Poor: 3 Intermediate: 2 Normal: 24 | 1 | Poor: 3 Intermediate: 1 Normal: 25 |
| - Size RV and LV | 15 | LV < RV: 0 LV = RV: 1 LV > RV: 14 | 2 | LV < RV: 0 LV = RV: 0 LV > RV: 27 *Missing: 1 |
| **IVC** | **8** |  | **7** |  |
| - Is there collapse? | 4 | Total collapse: 19 Some: 4 No or minimal: 3 | 4 | Total collapse: 7 Some: 16 No or minimal: 3 |
| - Collapsibility Index (CI) | 2 | Median: 47.3%  IQR: 37.9 – 55.9 | 3 | Median: 42.5% IQR: 33.3 – 62.2 |
| - What is the diameter? | 4 | Large: 4 Small: 17 Normal: 5 | 3 | Large: 5 Small: 13 Normal: 9 |
| - IVC diameter in mm | 8 | Median: 4.9 IQR: 3.8 – 6.4 | 8 | Median: 6.0  IQR: 3.9 – 7.9 |
| - IVC/Aorta ratio | 8 | Median: 0.73 IQR: 0.60 | 8 | Median: 0.77 IQR: 0.64 – 1.0 |
| **RUQ** | **8** |  | **0** |  |
| - Pleural effusion? | 0 | Yes: 0 No: 30 | 0 | Yes: 1 No: 29 |
| - Free fluid Morison’s pouch? | 1 | Yes: 0 No: 29 | 0 | Yes: 1 No: 29 |
| - Free perihepatic fluid? | 7 | Yes: 3 No: 20 | 0 | Yes: 1 No: 29 |
| **LUQ** | **2** |  | **1** |  |
| - Pleural effusion? | 0 | Yes: 0 No: 30 | 0 | Yes: 1 No: 29 |
| - Perisplenic free fluid? | 2 | Yes: 0 No: 28 | 0 | Yes: 0 No: 30 |
| - Free fluid splenorenal recess? | 0 | Yes: 0 No: 30 | 1 | Yes: 0 No: 29 |
| **Suprapubic/bladder** | **14** |  | **8** |  |
| - Free fluid posterior (transverse view) | 6 | Yes: 8 No: 16 | 4 | Yes: 4 No: 22 |
| - Free fluid posterior (longitudinal view) | 8 | Yes: 8 No: 14 | 4 | Yes: 3 No: 23 |
| **Right lung anterior** | **1** |  | **0** |  |
| - Lung sliding? | 1 | No: 2 Yes: 27 | 0 | No: 1 Yes: 29 |
| - B-lines? | 0 | White lung: 0 Confluent B-lines: 0 Yes, >3/ICS: 0 Yes, <3/ICS: 7 No: 23 | 0 | White lung: 0 Confluent B-lines: 0 Yes, >3/ICS: 1 Yes, <3/ICS: 0 No: 29 |
| **Right lung lateral** | **2** |  | **0** |  |
| - Lung sliding? | 2 | No: 2 Yes: 26 | 0 | No: 1 Yes: 29 |
| - B-lines? | 0 | White lung: 0 Confluent B-lines: 0 Yes, >3/ICS: 0 Yes, <3/ICS: 9 No: 21 | 0 | White lung: 0 Confluent B-lines: 0 Yes, >3/ICS: 0 Yes, <3/ICS: 0 No: 30 |
| **Left lung anterior** | **7** |  | **0** |  |
| - Lung sliding? | 5 | No: 1 Yes: 24 | 0 | No: 1 Yes: 29 |
| - B-lines? | 2 | White lung: 0 Confluent B-lines: 0 Yes, >3/ICS: 0 Yes, <3/ICS: 1 No: 27 | 0 | White lung: 0 Confluent B-lines: 0 Yes, >3/ICS: 0 Yes, <3/ICS: 0 No: 30 |
| **Left lung lateral** | **5** |  | **0** |  |
| - Lung sliding? | 5 | No: 0 Yes: 25 | 0 | No: 0 Yes: 30 |
| - B-lines? | 0 | White lung: 0 Confluent B-lines: 0 Yes, >3/ICS: 0 Yes, <3/ICS: 4 No: 26 | 0 | White lung: 0 Confluent B-lines: 0 Yes, >3/ICS: 0 Yes, <3/ICS: 0 No: 30 |

**Supplementary Table S3: p-RUSH views that were scored as "cannot interpret" by Reviewer 1 (R1) and Reviewer 2 (R2)**

| **Image^a^** | **R1 not interpretable^b^** n/N (%) | **R2 not interpretable^b^** n/N (%) |
| --- | --- | --- |
| **Total (32 x 30)** | 74/960 (7.7%) | 29/960 (3.0%) |
| **Cardiac views (14 x 30)** | 27/420 (6.4%) | 13/420 (3.1%) |
| **IVC views (2 x 30)** | 8/60 (13.3%) | 7/60 (11.7%) |
| **FAST (8 x 30)** | 24/240 (10.0%) | 9/240 (3.8%) |
| **Lung views(8 x 30)** | 15/240 (6.3%) | 0/240 (-) |

^a^ For every window, total number of questions x the number of patients is reported
^b^ Denominator is the number of scoring questions times the number of included patients
 Abbreviations: IVC = Inferior Vena Cava, FAST= Focused Assessment with Sonography in Trauma

**Supplementary Table S4: Type of shock reported by reviewer 1 and reviewer 2 and inter-observer variability.**

|  | **Reviewer 1 n/N** | **Reviewer 2**  **n/N** | **Inter-observer variability** |
| --- | --- | --- | --- |
| **Cardiogenic shock** | 3/23 | 1/27 | Kappa, unweighted = 0.225 (95% CI: 0.00; 0.510,  p-value=0.049)  Max. kappa: 0.483 |
| **Obstructive shock** | 0/23 | 0/27 |  |
| **Hypovolemic shock** | 15/23 | 10/27 |  |
| **Dissociative shock** | 2/23 | 4 |  |
| **Distributive shock** | 3/23 | 12/27 |  |

**Supplementary Table S5: Type of shock reported by reviewers combining hypovolemic and distributive shock, and inter-observer variability.**

|  | **Reviewer 1 n/N** | **Reviewer 2**  **n/N** | **Inter-observer variability** |
| --- | --- | --- | --- |
| **Cardiogenic shock** | 3/23 | 1/27 | Kappa, unweighted =  0.526 (95% CI: 0.230; 0.822,  p-value<0.001)  Max. kappa: 0.763 |
| **Obstructive shock** | 0/23 | 0/27 |  |
| **Hypovolemic/distributive shock** | 18/23 | 22/27 |  |
| **Dissociative shock** | 2/23 | 4 |  |

**
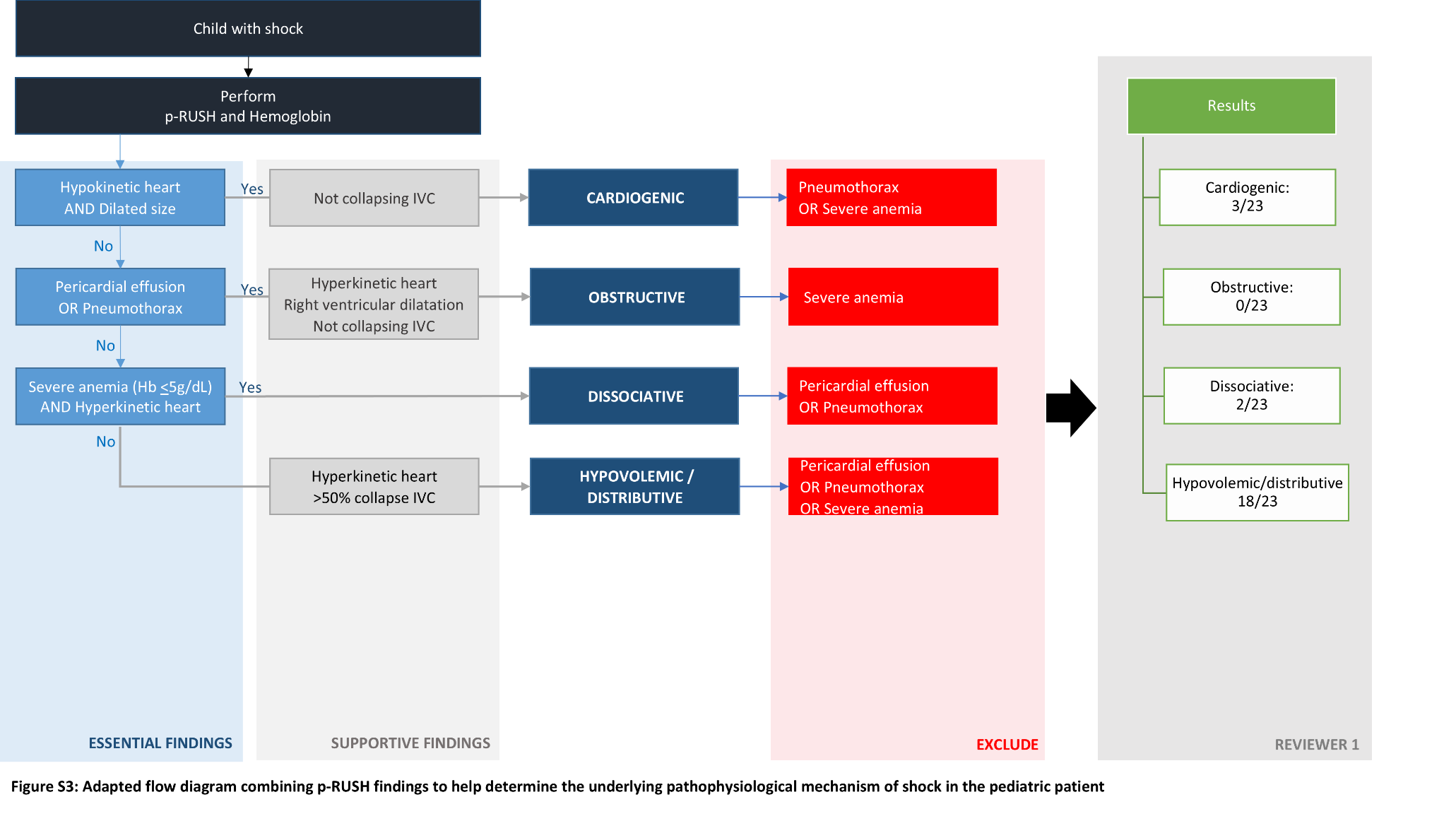
**
